# Acquired nephrogenic diabetes insipidus in a dog with leptospirosis

**DOI:** 10.1186/2046-0481-67-7

**Published:** 2014-04-17

**Authors:** Jamie L Etish, Peter S Chapman, Alan R Klag

**Affiliations:** 1 Veterinary Specialty and Emergency Center, Internal Medicine Service, 301 Veterans Highway, Levittown, PA 19056, USA

## Abstract

A 5 year old male neutered Cairn Terrier was evaluated for signs of polyuria and polydipsia. Initial hematology and chemistry panels were unremarkable and urinalysis showed a persistent hyposthenuria. Eleven days later, the dog became lethargic, inappetent and had developed acute renal failure. The dog was ultimately euthanized due to a poor response to treatment. Microscopic agglutination titres were consistent with a diagnosis of leptospirosis. The initial hyposthenuria in this case was consistent with acquired nephrogenic diabetes insipidus. This is an uncommon presentation of leptospirosis that has not previously been described to progress to acute renal failure. Leptospirosis should be considered as a differential diagnosis in any dog presenting with polyuria and polydipsia and these patients should be treated as a zoonotic risk.

## Background

Leptospirosis is a zoonotic disease caused by infection with pathogenic *Leptospira* spp*.*[1]*.* Clinical signs associated with leptospirosis can range from mild non-specific illness to severe disease associated with kidney, liver and lung injury. Polyuria and polydipsia are common findings in patients with leptospirosis [1]. The majority of infected dogs with polyuria and/or polydipsia are azotemic and the polyuria can be attributed to acute tubular injury, however up to 25% of dogs with dilute urine are not azotemic [2]. Some of these polydipsic, non-azotemic patients could have a decrease in renal function sufficient to impair concentrating ability without causing azotemia [3]. However, there is also evidence from experimental animal models that leptospires may cause a form of acquired nephrogenic diabetes insipidus (NDI) by making the collecting ducts resistant to the effects of vasopressin [4]. The patient in this report initially presented with signs compatible with acquired NDI that later progressed to acute renal failure (ARF). Although the latter syndrome is well-described, the former is only sporadically documented and the progression from NDI to ARF has not been previously described [3,5].

## Case presentation

A five year old male neutered cairn terrier was presented to the primary veterinarian for an acute onset of lethargy, vomiting, and inappetence. The dog had no travel history outside of Pennsylvania and was vaccinated as per American Animal Hospital Association recommendations, not including vaccination for leptospirosis [6]. A complete blood count (CBC) and serum biochemistry panel were performed with the significant abnormality being mild thrombocytopenia (Table 1). The dog was treated supportively with 20 ml/kg lactated Ringer’s solution^a^ subcutaneously. The dog’s clinical signs continued and it was presented to Veterinary Specialty and Emergency Center (VSEC) on day four. A physical examination revealed mild dehydration estimated at 5% without other abnormalities. An electrolyte panel was performed, revealing mild hypokalemia (Table 1). An abdominal ultrasound showed mild thickening of the gastric body and diffuse hyperechoic mucosal speckling of the small intestines. The patient was given supportive care for non-specific gastroenteritis with dolasetron^b^ (0.5 mg/kg intravenously [IV] q 24 h), famotidine^c^ (0.5 mg/kg IV q 24 h), metronidazole^d^ (10 mg/kg IV q 12 h), mirtazapine^e^ (7.5 mg per os [PO] q 24 h), and IV lactated Ringer’s solution at a rate of 4 ml/kg. The dog was discharged to the owner on day five with no further vomiting and an improved appetite. Treatment was continued at home with maropitant citrate^f^ (2.4 mg/kg PO q 24 h × 4 days), metronidazole^g^ (12.5 mg/kg PO q 12 h × 14 days), famotidine^h^ (0.5 mg/kg PO q 24 h × 7 days), mirtazapine (0.75 mg/kg PO q 24 h as needed for inappetance), and the patient was placed on a hydrolyzed diet^i^.

**Table 1:** Pertinent CBC and biochemistry findings

	**Day 1**	**Reference range for day 1**	**Day 4**	**Day 24**	**Day 29**	**Day 31**	**Reference range for days 4, 24, 29, 31**
Albumin (g/L)	32	23-40		33			27-44
Alkaline phosphatase (U/L)	166	23-212		173	133		5-131
Alanine aminotransferase (U/L)	29	10-100		55	27		12-118
Blood urea nitrogen (mmol/L)	2.5	2.5-9.6		2.86	19.6	23.6	2.1-11
Creatinine (μmol/L)	106.1	44.2-159.1		79.6	406.6	627.6	44.2-141.4
Glucose (mmol/L)	5.2	4.1-7.9	6.6	6.2			3.9-7.7
Cholesterol (mmol/L)	7.9	2.9-8.3		8. 9			2.4-8.4
Globulin (g/L)	42	25-45		40			16-36
Total bilirubin (μmol/L)	5.1	0-15.4		3.4			1.7-5.1
Phosphorous (mmol/L)	1.3	0.8-2.2		0.7		2.8	0.8-2.2
Potassium (mmol/L)	3.9	3.5-5.8	3.2	3.2	3.1		3.6-5.5
Sodium (mmol/L)	147	144-160	148	142	139		139-154
Chloride (mmol/L)	111	109-122	99	100	99		102-120
Hematocrit (Proportion of 1.0)	0.47	0.37-0.55		0.41			0.36-0.60
White blood cells (× 10^9^/L)	6.84	5.5-16.9		14.2			4.0-15.5
Neutrophils (× 10^9^/L)	5.96	2-12		10.79			2.06-10.6
Bands (× 10^9^/L)				0			0-0.3
Eosinophils (× 10^9^/L)	0.05	0.1-1.49		0.142			0-1.200
Lymphocytes (× 10^9^/L)	0.34	0.5-4.9		2.27			0.69-4.50
Platelets (× 10^9^/L)	139	175-500		160			170-400

On day 24, the dog was presented to VSEC for re-evaluation after the owner noted an increased thirst and inappropriate urination in the house since day 18, without hematuria, stranguria, or pollakiuria. There was no recurrence of the gastrointestinal signs and the dog’s appetite remained good. Physical examination was unremarkable. A CBC showed persistent mild thrombocytopenia. A serum biochemistry panel showed mild increases in alkaline phosphatase, cholesterol and globulins, mild hypokalemia, and other non-specific abnormalities as shown in Table 1. Urine was hyposthenuric (urine specific gravity [USG] 1.002) with trace protein on urine dipstick (Table 2) and an unremarkable urine sediment. A urine culture was submitted to rule out an occult urinary tract infection.

**Table 2:** Urinalysis results

	**Day 24**	**Day 26**	**Day 28**	**Day 29**	**Normal reference range**
Urine specific gravity	1.002	1.006	1.004	1.006	1.015-1.050
pH	7.0			6.0	5.5-7.0
Protein	Trace			Negative	Negative
Glucose	Negative			Negative	Negative
Ketone	Negative			Negative	Negative
Bilirubin	Negative			Negative	Negative to 1+
Blood	Negative			3+	Negative
WBC	Negative			2-3	0-3/HPF^a^
RBCS	Negative			0-1	0-3/HPF
Casts	None			None	
Crystals	None			None	
Bacteria	None			None	None
Transitional cells	None			0-3	0-1/HPF
Squamous cells	None			0-1	0-3/HPF

^a^HPF = High power field.

Given the history, marked hyposthenuria, and absence of azotemia and hyperglycemia, psychogenic (primary) polydipsia, polydipsia secondary to gastrointestinal disease, and diabetes insipidus were amongst the primary differentials [7]. Polydipsia secondary to gastrointestinal disease was considered unlikely given the lack of resolution with treatment of the gastrointestinal signs [7]. Renal insufficiency was excluded at that time based on the presence of hyposthenuria, suggesting active dilution of the urine [8]. The owner was asked to collect a series of at-home urine samples from the dog over the following week to evaluate for evidence of any spontaneous concentrating ability that would support a diagnosis of psychogenic polydipsia.

The dog was re-evaluated on day 29. The polyuria had persisted but the dog was now lethargic, inappetent, was intermittently vomiting, and had had a significantly reduced thirst over the past 5 days. On physical examination, tacky mucous membranes and decreased skin turgor were noted; based on these findings and a decrease in weight from 9.5 kg to 8.6 kg, the dog was estimated to be 10% dehydrated. No other new physical examination findings were noted. A serum biochemistry panel revealed moderate azotemia (Table 1). Urine specific gravity was 1.006 on a urinalysis with 2+ protein and 3+ blood (Table 2). Abdominal ultrasound revealed mild to moderate bilateral renomegaly (left kidney measured 5.8 cm in craniocaudal dimension versus 5.3 cm on day 4; right - 6.1 cm versus 4.8 cm). The kidneys had a rounded, swollen appearance with increased echogenicity of the medullae. The liver was also mildly enlarged and diffusely hypoechoic. The ultrasonographic changes to the gastrointestinal tract persisted, but were improved compared to day 4. Resting cortisol was 6.1 μg/dL, excluding a diagnosis of hypoadrenocorticism [9]. The urine protein: creatinine ratio was 4.5. The urine culture from day 24 showed no bacterial growth. Serum was submitted for microscopic agglutination test (MAT) against a panel of seven leptospiral serogroups.

The patient was admitted to the hospital, and treatment with IV lactated Ringer’s solution, ampicillin^j^ (20 mg/kg IV q 8 hr), doxycycline^k^ (10 mg/kg IV q 24 hr), dolasetron (0.5 mg/kg IV q 24 hr), and famotidine (0.5 mg/kg IV q 24 hr) was initiated. An indwelling urinary catheter was placed to measure urine output and to protect hospital staff and clients against exposure to potentially infectious leptospires in the urine. On day 31, the dog had a decline in urine output (from 3.1 ml/kg/hr on day 30 to 1.8 mls/kg/hr on day 31) and developed hypertension (200 mmHg systolic measured by noninvasive doppler). The azotemia had significantly worsened (Table 1). The urine output did not increase after boluses of furosemide^l^ (1 mg/kg IV) and mannitol^m^ (0.5 g/kg IV twice) and the owners elected to have the dog euthanized. The MAT results were received after the dog was euthanized and showed high titres against multiple serovars (Table 3). Given the clinical signs and absence of vaccination against leptospirosis, the markedly elevated MAT in this case was considered to confirm a diagnosis of leptospirosis [1,10]. Since multiple serovars had a titre >1:3200, the causative serovar could not be determined.

**Table 3:** Microscopic agglutination test results

**Leptospirosis species and serovar**	**Titer value**
*L. interrrogans* serovar Pomona	Positive 1:3200
*L. interrrogans* serovar Icterohaemorrhagia	Negative </=1:50
*L. interrrogans* serovar Canicola	Negative </=1:50
*L. kirschneri* serovar Grippotyphosa	Positive >1:6400
*L. interrrogans* serovar Hardjo	Negative </=1:50
*L. interrrogans* serovar Autumnalis	Positive 1:6400
*L. interrrogans* serovar Bratislava	Positive 1:3200

## Discussion

The progression of leptospirosis in this case did not follow the typical pattern that is expected in this disease [3]. The initial polyuria and polydipsia were accompanied by hyposthenuria and normal biochemical parameters. Although leptospirosis has been reported to cause isosthenuria or hyposthenuria without azotemia, the authors of these previous studies did not report whether these cases progressed to more severe signs of renal injury [2,5,11]. Typically, renal insufficiency is considered less likely if the urine specific gravity is less than 1.006 [8]. However, hyposthenuric urine can be present in renal failure but suggests the presence of another disease process that is leading to the hyposthenuria – in this case this may have been the presence of leptospires in the renal tubules [12].

Causes of polyuria and polydipsia can be categorized as primary polydipsia, osmotic diuresis, central diabetes insipidus (CDI), congenital NDI or acquired NDI. Acquired NDI can be caused by various conditions such as hypoadrenocorticism, hyperadrenocorticism, pyometra, pyelonephritis and hypercalcemia which may impair urinary concentrating ability [8]. In this patient, there was no evidence of an osmotic diuresis; CDI was considered unlikely since serum sodium was normal in the face of significant dehydration; and primary polydipsia was considered unlikely since there was no evidence of any concentrating ability after a significant decrease in water intake over five days [8]. The five days of decreased water intake should have corrected for any medullary washout, exceeding the number of days recommended during a water deprivation test [8]. Other causes of acquired NDI were considered unlikely based on presenting complaints and signalment (e.g. hyperadrenocorticism) or excluded based on diagnostic findings (e.g. hypoadrenocorticism, hypercalcemia, urinary tract infection). As a result, the authors consider the initial polydipsia in this case to be consistent with acquired nephrogenic diabetes insipidus due to decreased responsiveness of the collecting ducts to vasopressin in the presence of leptospirosis [4]. Though this dog may have responded to more aggressive treatment, such as continued fluid therapy guided by measurement of central venous pressures or performing dialysis, the owners elected for euthanasia and declined a post-mortem examination. Although a post-mortem examination was not performed, markedly elevated MAT titres against several leptospiral serogroups in an unvaccinated patient are highly indicative of acute infection with *Leptospira*. In addition, this patient had a persistent mild thrombocytopenia, which increases the suspicion for the diagnosis of leptospirosis in an azotemic patient [3].

Leptospirosis is a well-established zoonotic disease [3]. Transmission to humans usually occurs from chronic carrier states rather than from incidental hosts that develop acute leptospirosis [13,14]. Leptospires have been shown to be present in urine prior to the initiation of proper treatment and in the first two to three days of treatment in rodent models [15]. It has been recommended that appropriate protective precautions should be taken in all dogs with acute renal failure until leptospirosis is excluded or an alternate diagnosis is reached [3]. Although it is possible that the initial clinical signs of gastritis and inappetence in this patient were caused by an unrelated disease, they may have been due to early leptospirosis [2,16,17]. Since leptospires are shed in the urine within 7-10 days after infection, it is therefore very likely that this patient was shedding organisms on day 24 when first evaluated for polyuria and polydipsia [3]. Appropriate precautions to protect against the zoonotic potential of leptospirosis were not taken at this time. In fact, the owners were instructed to collect urine samples at home, which could have exposed them to infectious organisms. The authors would recommend that the same handling precautions that are taken with the urine of patients with acute renal failure are also followed in patients with polyuria and polydipsia until an alternate final diagnosis is reached.

## Conclusion

Leptospirosis should be considered a differential diagnosis in any patient presented with polyuria and/or polydipsia without another apparent underlying cause. These patients should be monitored closely for progression to azotemia and should be treated as a zoonotic risk. Given the potential for progression to acute renal failure, the authors recommend routine screening for leptospirosis before undertaking procedures such as a water deprivation test that may place the patient at additional risk for renal injury [8]. In conclusion, this case presents evidence that leptospirosis can cause acquired NDI and can progress to ARF in some patients.

## Endnotes

^a^Lactated Ringer’s solution; Abbott Laboratories, North Chicago, IL.

^b^Anzemet; Sanofi-Aventis U.S., LLC, Bridgewater, NJ.

^c^Famotidine; Baxter Healthcare Corp., Deerfield, IL.

^d^Metronidazole; Hospira, Inc., Lake Forest, IL.

^e^ Remeron; Aurolife Pharma, LLC, Dayton NJ.

^f^Cerenia; Pfizer Animal Health, New York, NY.

^g^Metronidazole; Teva Pharmaceuticals USA, Inc., Sellersville, PA.

^h^Famotidine; Wockhardt Limited, Mumbai, India.

^i^Royal Canin HP diet; Royal Canin USA, Inc, St Charles MO.

^j^Ampicillin; Sagent Pharmaceuticals Inc., Schaumburg, IL.

^k^Doxycycline; APP Pharmaceuticals, LLC., Schaumburg, IL.

^l^Salix; Intervet, Inc., Millsboro, DE.

^m^Mannitol; Nova-Tech, Inc., Grand Island, NE.

## Abbreviations

ARF: Acute renal failure; CBC: Complete blood count; CDI: Central diabetes insipidus; IV: Intravenous; NDI: Nephrogenic diabetes insipidus; MAT: Microscopic agglutination test; PO: Per os; USG: Urine specific gravity; VSEC: Veterinary Specialty and Emergency Center.

## Competing interests

The authors declare that they have no competing interests.

## Authors’ contribution

JE gathered case material, supportive evidence, and drafted the manuscript. PC and AK participated in providing supportive evidence and editing. All authors read and approved the final manuscript.
